# Surgical management of Rathke cleft cysts in pediatric patients: a single institution experience

**DOI:** 10.1007/s00381-024-06277-z

**Published:** 2024-01-19

**Authors:** Michael G. Brandel, Christine Lin, Robert C. Rennert, Jillian H. Plonsker, Usman A. Khan, John R. Crawford, Javan Nation, Michael L. Levy

**Affiliations:** 1 Department of Neurosurgery, University of California, San Diego-Rady Children’s Hospital, San Diego, CA 92123 USA; 2 Department of Neurosurgery, University of Utah, Salt Lake City, UT 84112 USA; 3 Division of Child Neurology and Neurosciences Institute, Children’s Hospital of Orange County and University of California Irvine, Orange, CA 92868 USA; 4 Division of Neurology, Rady Children’s Hospital, San Diego, CA 92123 USA; 5 Department of Otolaryngology, University of California, San Diego-Rady Children’s Hospital, San Diego, CA 92123 USA

**Keywords:** Resection, Transsphenoidal, Orbitozygomatic, Pituitary

## Abstract

**Objective:**

Rathke cleft cysts (RCCs) are benign, epithelial-lined sellar lesions that arise from remnants of the craniopharyngeal duct. Due to their rarity in the pediatric population, data are limited regarding the natural history and optimal management of growing or symptomatic RCCs. We present our institutional experience with the surgical management of RCCs.

**Methods:**

We performed a retrospective study of consecutive RCC patients ≤ 18 years old treated surgically at our institution between 2006 and 2022.

**Results:**

Overall, 567 patients with a diagnosis of pituitary mass or cyst were identified. Of these, 31 had a histopathological diagnosis of RCC, 58% female and 42% male. The mean age was 13.2 ± 4.2 years. Presenting symptoms included headache (58%), visual changes (32%), and endocrinopathies or growth delay (26%); 13% were identified incidentally and subsequently demonstrated growth on serial imaging. Six percent presented with symptomatic intralesional hemorrhage. Surgical approach was transsphenoidal for 90% of patients and orbitozygomatic for 10%. Preoperative headaches resolved in 61% of patients and preoperative visual deficits improvement in 55% after surgery. New pituitary axis deficits were seen in 9.7% of patients. Only two complications occurred from a first-time surgery: one cerebrospinal fluid leak requiring lumbar drain placement, and one case of epistaxis requiring cauterization. No patients experienced new visual or neurological deficits. Patients were followed postoperatively with serial imaging at a mean follow-up was 62.9 ± 58.4 months. Recurrence requiring reoperation occurred in 32% of patients. Five-year progression-free survival was 47.9%. Except for one patient with multiple neurological deficits from a concurrent tectal glioma, all patients had a modified Rankin Scale score of 0 or 1 (good outcome) at last follow-up.

**Conclusion:**

Due to their secretory epithelium, pediatric RCCs may demonstrate rapid growth and can cause symptoms due to local mass effect. Surgical management of symptomatic or growing pediatric RCCs via cyst fenestration or partial resection of the cyst wall can be performed safely, with good neurologic outcomes. There is a nontrivial risk of endocrinologic injury, and long-term follow up is needed due to high recurrence rates.

## Introduction

Rathke cleft cysts (RCCs) are benign, epithelial-lined sellar or suprasellar lesions that arise from remnants of the craniopharyngeal duct. RCCs occur when Rathke’s cleft, found in the pars intermedia of the pituitary gland, fails to regress around the eighth week of gestation. The cleft evolves into a cyst when fluid accumulates.

RCCs can be identified among all age groups but are most commonly found between ages 40 and 60 and less commonly found in children. Prevalence in the adult population has been reported at 12–33%, compared to 1.2–3.0% in the pediatric population [1–4].

On computed tomography (CT), RCCs can appear as hypodense, isodense, or hyperdense homogeneous lesions, and ring-like enhancement can be seen in up to 50% of cysts [5]. Appearance on magnetic resonance imaging (MRI) varies depending on cyst contents: clear fluid is hypointense on T1 and hyperintense on T2, while protein-rich, mucous fluid is hyperintense on both T1 and T2 [6]. Blood in RCCs may appear hyperintense on both T1 and T2. Additionally, up to 77% of RCCs contain an intracystic, solid nodule that appears hyperintense on T1 and hypointense on T2 [7–9]. RCCs can be differentiated from other supra- and intrasellar masses such as pituitary adenomas and craniopharyngiomas by their ovoid shape and minimal or lack of cyst wall enhancement [9]. In contrast to adenomas, RCCs are located in the midline [1].

On histopathology, cyst walls typically consist of secretory epithelium that is either pseudostratified ciliated cuboidal or columnar. Squamous metaplasia is sometimes present, as are goblet cells and cholesterol clefts. Cyst wall epithelial cells have a higher Ki-67 protein content, a cellular marker for proliferation, as compared to epithelial cells elsewhere in the cyst [9, 10]. Additionally, RCCs express different cytokeratins compared to craniopharyngiomas, allowing for differentiation of diagnosis—this is notable given that RCCs and craniopharyngiomas are otherwise considered along the same histopathologic spectrum.

Analysis of routine autopsies revealed that 13–33% of normal pituitary glands harbor RCCs [9]. Those that progress can cause symptoms due to mass effect, especially when suprasellar. Common presenting symptoms include headaches (55–81%), endocrinopathies (46–81%), and visual deficits (34–75%) [6, 11]. In the literature, gonadotropin deficiency is the most common endocrinopathy (60%), followed by ACTH and TSH deficiencies (both 36%), and hyperprolactinemia (31%) [12]. GH deficiency can be seen in 12–79% of cases [9]. Mass effect on the posterior pituitary gland can cause DI in up to 37% of adult patients, a rate that surpasses that seen in pituitary adenomas [1]. Pituitary apoplexy is uncommon but can occur, presenting with sudden, severe headache, visual disturbances, pituitary dysfunction, and altered mental status [10].

The advent of advanced imaging techniques has allowed for increased incidental detection of RCCs, especially in asymptomatic children. Surgical intervention is not usually indicated for asymptomatic, incidental cysts, and consequently without histological information, it is impossible to confirm the diagnosis of RCC. Symptomatic RCCs are typically treated with surgical drainage or resection, using either a transsphenoidal or transcranial approach. However, the secretory nature of RCC epithelium lends itself to recurrence following surgical treatment, with rates nearing 50% when follow-up periods extend over 4 years and recurrence most commonly occurring within the first 6 years after surgery [5, 12–15].

Due to their rarity in children, data are limited regarding the natural history and management of growing or symptomatic RCCs. This study focuses on RCCs requiring surgical intervention with histopathological confirmation of diagnosis, and represents the largest pediatric case series of RCCs requiring surgery to date [6, 8, 9, 11, 13, 14, 16, 17].

## Methods

Institutional Review Board (IRB) approval was obtained, and a retrospective chart review was performed for RCC patients ≤ 18 years old treated between 2006 and 2022 at Rady Children’s Hospital San Diego (RCHSD). We reviewed the electronic medical record of 567 patients with a diagnosis of pituitary mass or cyst, and included only those who underwent surgical management of their lesion with a histopathological diagnosis of RCC. We collected demographic information, symptoms present at diagnosis, cyst size and features, surgical approach, post-operative complications, endocrinologic outcomes, and post-operative symptoms. Counts and percentages are reported. Kaplan Meier curves were used to calculate progression-free survival. Statistical analyses were performed with Stata MP Version 14.1 (Stata Corp LP).

### Surgical strategy

Common surgical indications for surgery included symptoms of mass effect such as headache, visual field deficits, and endocrinopathies, and growth on serial imaging. At RCHSD symptomatic RCCs are treated preferentially via an endoscopic-assisted transsphenoidal approach in conjunction with otolaryngology, with transcranial approaches reserved for larger lesions not amenable to transsphenoidal surgery. All transsphenoidal approaches since 2015 have involved an endoscopic-assisted approach, whereas beforehand the majority were microscopic sublabial approaches. Treatment of the cyst involves fenestration, complete cyst drainage with marsupialization, and partial wall removal (as deemed safe).

## Results

### Patient characteristics

Between 2006 and 2022, 31 children with pituitary lesions underwent surgery and had a histopathological diagnosis of RCC (Tables 1 and 2). Fifty-eight percent were female and 42% were male. The mean age was 13.2 ± 4.2 years. The most common presenting symptoms were headache (58%), visual changes (32%), and endocrinopathies or growth delay (26%). Specifically, 10% of patients presented with diabetes insipidus (DI), 6% with adrenocorticotropic hormone (ACTH) deficiency, 6% with growth hormone (GH) deficiency, 6% with precocious puberty, 3% with thyroid stimulating hormone (TSH) deficiency, 3% with gonadotropin deficiency, and 3% hyperprolactinemia. Thirteen percent of RCCs were identified incidentally and grew significantly on serial imaging; 6% presented with symptomatic intralesional hemorrhage.

**Table 1:** Patient demographic information

**Factor**	***N* (%)**
Gender	-
Female	18 (58%)
Male	13 (42%)
Age	13.2 +/− 4.2
Ethnicity	-
Asian	4 (13%)
Black	1 (3%)
Caucasian	8 (26%)
Hispanic or Latino	18 (58%)
Presenting symptoms	
Headache	18/31 (58%)
Visual disturbance	11/31 (35%)
Endocrinopathy or growth delay	8/31 (26%)
* Gonadotropin deficiency*	*1/31*
* ACTH deficiency*	*2/31*
* TSH deficiency*	*1/31*
* GH deficiency*	*2/31*
* Hyperprolactinemia*	*1/31*
* Diabetes insipidus*	*3/31*
* Precocious puberty*	*2/31*
Incidental finding	4 (13%)
Surgical approach	
Transsphenoidal	28 (90%)
Orbitozygomatic	3 (10%)

**Table 2:** Patient clinical presentation

Case #	Age at surgery (years), sex	Headache	Visual disturbance	Endocrinopathy	Incidental finding	Indication for surgery
1	10, F	-	+	+	-	VFD, precocious puberty
2	1, M	-	+	-	-	Optic nerve pallor
3	15, F	+	-	-	-	Headache
4	14, F	+	-	-	-	Headache
5	12, F	-	-	+	-	DI
6	6, M	+	-	-	-	Headache
7	16, F	+	-	-	-	Headache, decreased visual acuity
8	11, M	-	+	-	-	Decreased visual acuity
9	16, M	+	-	-	-	Headache
10	17, F	+	-	-	-	Headache, elevated testosterone / androstenedione
11	18, F	+	-	-	-	Headache
12	11, F	-	-	+	-	Precocious puberty, growth on serial imaging
13	16, M	+	-	+	-	GH deficiency
14	15, M	+	+	-	-	Headache, VFD
15	19*, M	-	-	-	+	Growth on imaging
16	16, F	-	+	-	-	Decreased visual acuity
17	13, M	-	+	-	-	Decreased visual acuity
18	11, F	-	-	+	-	Panhypopituitarism
19	12, M	+	-	-	-	Headache
20	4, M	-	-	-	+	Large size, mass effect, need for pathologic diagnosis
21	15, M	+	-	+	-	Headache, DI
22	8, F	-	-	-	+	Growth on imaging
23	14, F	-	+	+	-	Impaired peripheral vision, GH/ACTH/LH/FSH/ TSH deficiencies
24	15, F	+	+	-	-	Headache, decreased visual acuity
25	7, F	+	+	-	-	Headache, CN palsy
26	18, F	+	-	-	-	Headache
27	16, F	-	-	-	+	Growth on imaging
28	16, F	+	-	+	-	Headache, hyperprolactinemia
29	14, M	+	-	-	-	Headache
30	8, M	+	-	-	-	Headache
31	12, F	+	+	-	-	Headache, abnormal eye movements

*DI* diabetes insipidus, *VFD* visual field deficit

*Patient was diagnosed on imaging at age < 18

Nine patients (29%) in our series were classified as having isolated headache (headache in the absence of endocrinopathy, visual disturbance, or another neurological deficit). These lesions were considered for surgery on a case-by-case basis in a multidisciplinary fashion with the endocrinology, ophthalmology, and neuro-oncology services. Common factors that supported surgical intervention in these patients included mass effect on the optic chiasm or pituitary gland, solid components concerning for adenoma or craniopharyngioma, and intralesional hemorrhage plus sudden-onset headache concerning for apoplexy. The mean RCC size overall was 13.7 ± 6.6 mm and was significantly larger for patients presenting with isolated headache (18.3 vs. 11.7 mm, *p* = 0.008).

### Management and outcomes

Overall, 90% underwent a transsphenoidal approach (17 endoscopic, 11 microscopic), and 10% underwent an orbitozygomatic approach. Intraoperative CSF leaks were encountered in 15 of 28 (54%) transsphenoidal cases. Two were high-flow CSF leaks and the remainder were low flow leaks. We utilized a nasoseptal flap in only one case. Reconstruction techniques for the remainder involved some combination of acellular dermal matrix, gelfoam, free mucosal graft, and abdominal fat graft. Intraoperative lumbar drains were placed in 4 of 15 (27%) cases in which CSF leaks were encountered. There were no intraoperative complications.

### Postoperative care

Following RCC surgery, all RCHSD patients were admitted to the pediatric intensive care unit (PICU) for surveillance. Patients were managed concurrently by the PICU, neurosurgical, and endocrinology teams, with particular attention to any emergence or changes in endocrinopathies and/or DI. Postoperative MRIs with and without contrast were typically performed within 48 h of surgery.

The average length of hospitalization was 5.4 days (range: 2–14). Thirteen patients (42%) developed postoperative DI, half of which resolved by discharge from the hospital. Three patients (9.7%) developed postoperative pituitary axis deficiencies (Table 3); one (1/3, 33%) after a transcranial approach, two (1/11, 9%) after a microscopic transsphenoidal approach, and none (0/17, 0%) after an endoscopic transsphenoidal approach.

**Table 3:** Pre- and postoperative endocrinopathies

**Endocrinopathy**	**Preoperative**	**Postoperative**			
	*N* (%)	Improved	Persisted	New onset, transient	New onset, permanent
		*N*	*N*	*N*	*N*
ACTH deficiency	2 (6)	1	1	1	3
Diabetes insipidus	3 (10)	1	2	6	7
GH deficiency	2 (6)	1	1	0	3
Gonadotropin deficiency	1 (3)	0	1	0	3
Hyperprolactinemia	1 (3)	0	1	0	0
Precocious puberty	2 (6)	2	1	0	0
TSH deficiency	1 (3)	0	1	0	5

Patients were followed postoperatively with surveillance MRIs along with clinical visits, with a mean follow-up of 62.9 ± 58.4 months. No patients were lost to follow-up. Preoperative headaches resolved in 61% of patients after surgery overall (and 78% of those presenting with isolated headache), and 55% of patients with preoperative visual deficits reported improvement following surgery. Only two postoperative complications occurred from a first-time surgery: one cerebrospinal fluid (CSF) leak requiring lumbar drain placement and one case of epistaxis requiring cauterization. No patients (0%) experienced new visual or neurological deficits.

**Fig. 1:**
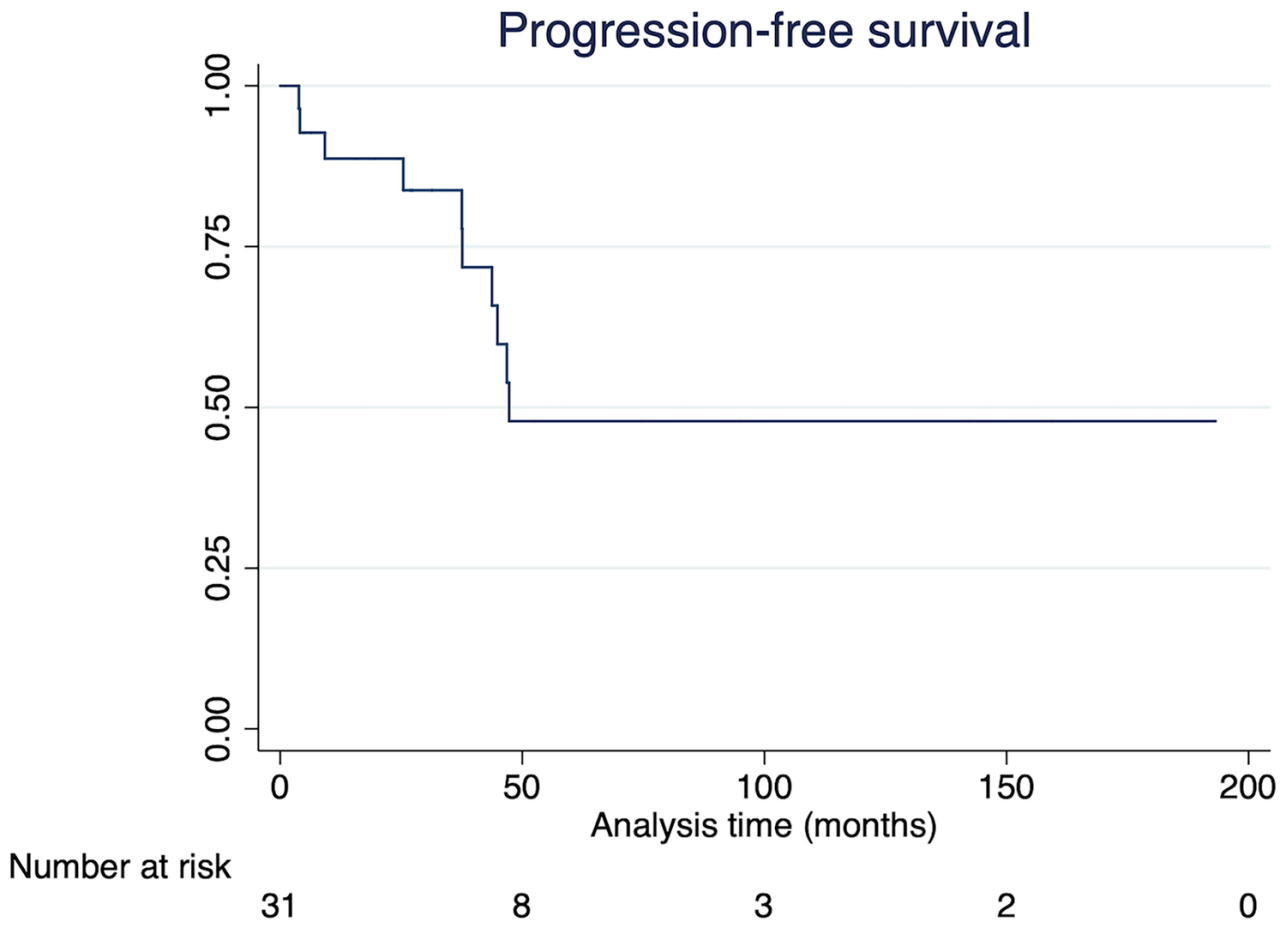
Kaplan-Meier estimation of progression-free survival

Recurrence requiring reoperation occurred in 32% of patients (*N* = 10) with a median time to recurrence of 37.6 months (interquartile range [IQR] 9.2–44.9 months) and occurring within 5 years for all ten (100%) patients. Recurrence was asymptomatic for 80% of patients and symptomatic for 20% of patients (headache for one patient and peripheral vision loss in one patient). Nine patients (90%) underwent the same approach for their revision surgery as their original surgery. Surgical approach for the first reoperation was 80% transsphenoidal and 20% pterional. One patient underwent a second reoperation via transsphenoidal approach after his RCC recurred again; both of his prior surgeries were via the transsphenoidal approach. Five-year progression-free survival was 47.8% (Fig. 1). Except for one patient with multiple neurological deficits from a concurrent tectal glioma, all patients had a modified Rankin Scale (mRS) score of 0 or 1 at their last follow-up visit.

### Endocrinologic outcomes

Following resection, four out of the eight patients (50%) experienced resolution of their endocrinopathies. Seven patients (23%) did experience persistent DI.

### Patient example

**Fig. 2:**
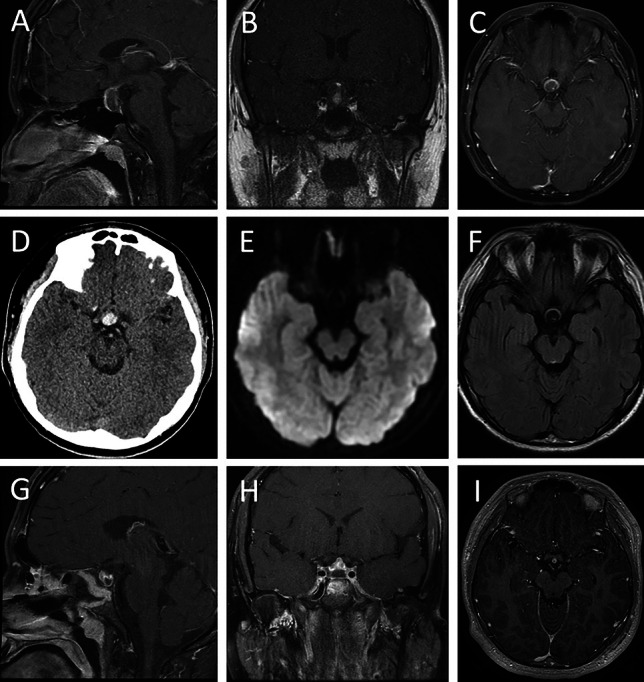
A 14-year-old boy with hemorrhagic RCC. **A** Sagittal, **B** coronal, and **C** axial views of a T1 post-contrast MRI demonstrating a trans-sellar structure measuring 12 × 16 mm with T1 hyperintensity and mildly thickened peripheral enhancement. The lesion abuts the undersurface of the optic chiasm. The stalk enhances normally. The optic chiasm is slightly bowed superiorly. It is hyperdense on CT (**D**) with Hounsfield units of approximately 70, consistent with hemorrhage. It is not diffusion restricting on DWI (**E**) and it is hypodense on T2 (**F**). Postoperative **G** sagittal, **H** coronal, and **I** axial views of a T1 post-contrast MRI show postsurgical changes of transsphenoidal resection within the nasal passages, paranasal sinuses and the sella itself with residual enhancing tissue which likely represents pituitary adenohypophysis

A 14-year-old previously healthy male presented with an acute-onset severe headache and vomiting. Imaging demonstrated a sellar/suprasellar hemorrhage concerning for pituitary apoplexy with bowing of the optic chiasm (Fig. 2). There was no visual change or endocrinologic disturbance. A transsphenoidal endoscopic resection was performed, yielding a green mucoid and fibrous lesion. This was resected in a piecemeal fashion. Pathology was consistent with RCC. He received a follow-up MRI within 48 h which revealed decompression of the cyst with resolution of mass effect on the chiasm. The patient had DI postoperatively and was discharged on desmopressin, which he was ultimately able to discontinue. His headache and vomiting resolved, and he did not develop any vision or endocrinologic issues. At 1-year post-op, he remained at his neurological baseline with no recurrence of his symptoms.

## Discussion

**Fig. 3:**
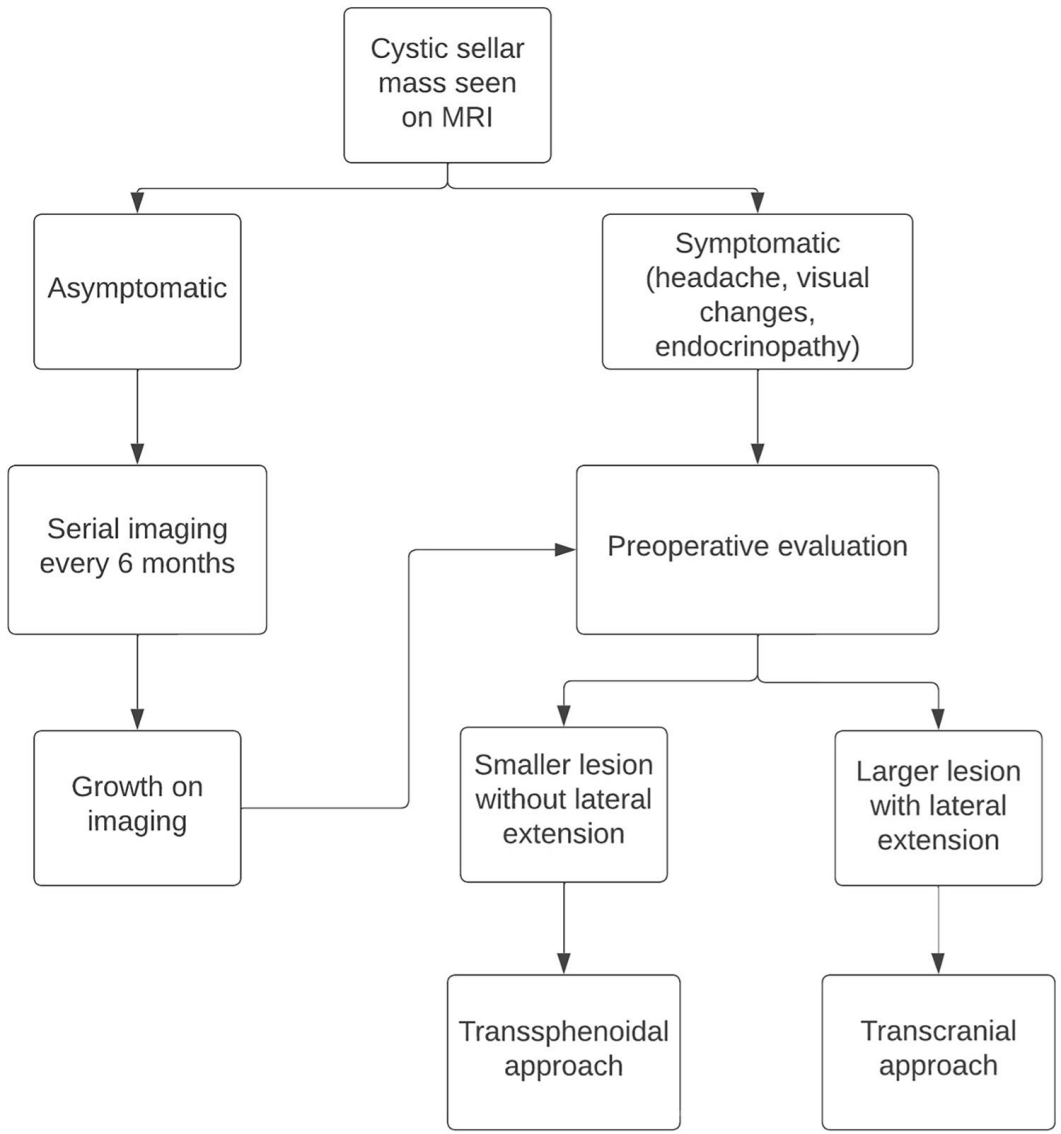
Decision tree utilized for evaluation of surgical candidacy

Given that many cystic intracranial lesions including RCCs are benign and do not require surgical intervention, careful consideration is warranted prior to offering surgical management. Prior studies have delineated factors that necessitate surgery [9] and treatment selection algorithms exist for benign intracranial cysts [18], but not yet for RCCs. At our center, surgical decision-making is guided by presence of symptoms interfering with quality of life and lesion growth on serial imaging. Asymptomatic patients are followed via serial imaging every 6 months; patients with significant growth on serial imaging were referred for presurgical evaluation (Fig. 3). Significant growth is defined as consistent growth over time. Three patients (10%) in our cohort experienced consistent growth over serial imaging and thus were offered surgery. Symptomatic patients were evaluated for surgery if they experienced medically refractory headaches, visual field deficits diagnosed via formal ophthalmological evaluation, or endocrinopathies necessitating medical intervention.

In this series, headache was the most common presenting symptom, followed by visual disturbances and endocrinopathies, consistent with the presenting symptoms reported in the adult literature. Most patients with headaches experienced resolution following surgery. Of the ten patients with visual disturbances, four (40%) presented with decreased visual acuity, two with visual field deficits (20%), one with impaired peripheral vision, one with cranial nerve palsy, one with impaired peripheral vision, and one with abnormal eye movements. Half of the patients with preoperative visual deficits experienced postoperative improvement, which has implications for future independence. While RCCs are histopathologically benign, the associated symptoms can significantly impact patients’ quality of life and affect their education; visual field deficits preclude patients from driving in some states and countries, precipitating long-term detriments to their quality of life.

Over a quarter of the patients in our cohort presented with preoperative endocrinopathies, of which DI was the most common. New onset DI was also frequently encountered after surgery and was persistent in 23% of patients including the nearly 10% who developed panhypopituitarism. Prior pediatric literature has reported a similar postoperative DI incidence with notably higher rates of persistent DI in children compared to adults, with younger age identified as the primary risk factor [16, 19]. While this rate is not insignificant, this finding potentially reflects patient selection given our high threshold to operate and that postoperative endocrinopathies may be higher risk in patients with chronic mass effect on their pituitary gland. Furthermore, subgroup analysis revealed a 0% rate of postoperative panhypopituitarism among patients who underwent endoscopic transsphenoidal approaches (*n* = 17). Given the overall low rate of surgical complications in our cohort, along with frequent improvement in symptoms related to mass effect, our data support surgical intervention for pediatric RCC when symptoms arise.

Symptomatic RCCs are typically treated with surgical drainage, resection, or marsupialization. Pediatric populations have few medical contraindications to surgery. There are no alternative treatments for RCCs. Historical surgical management involved cyst wall fenestration to reduce mass effect, which involves the creation of a small opening in the anterior pituitary gland into the cyst cavity, but this approach may yield higher recurrence rates compared to complete resection. However, evidence is mixed with a wide range of recurrence rates reported across the literature [6, 8, 9, 11, 13, 14, 16, 17]. Complete resection, which includes extracapsular dissection with decompression of the cyst and removal of the cyst wall, may also be associated with higher rates of post-operative endocrinological sequelae [18, 20]. Marsupialization is an increasingly employed technique that entails a wide opening in the anterior cyst wall which decompresses the cyst and provides a dependent drainage pathway without risking injury to surrounding structures.[21] A meta-analysis of 1151 surgical RCC patients of all ages showed that the transsphenoidal approach was most commonly employed (96%), while a transcranial approach was less frequently used (4%) [11]. In our series, transsphenoidal approaches were employed in 90% of cases, as this approach is less invasive as compared to transcranial approaches, and has essentially no cosmetic impact. In the pediatric population, however, this approach can at times be challenging due to smaller-sized nares, necessitating turbinate or septum resection and increasing risk of postoperative empty nose syndrome. Awareness of the stepwise pneumatization of the pediatric sinus and anterior skull base growth patterns during pediatric development is also critical, as they can affect intranasal bony surgical landmarks and traditional operative [22, 23]. Our center’s transsphenoidal cases are nonetheless performed in conjunction with otolaryngology, with significant combined expertise in pediatric anterior skull base approaches [19, 22, 24, 25]. Larger lesions with lateral extension are approached transcranially to allow for an optimal window of resection. Accordingly, no patients in our cohort experienced significant intraoperative surgical or postoperative rhinological complications.

Despite the advantages of the transsphenoidal approach, larger cysts with significant suprasellar (including cysts that extend into the third ventricle and threaten CSF flow) or lateral extension can sometimes necessitate a transcranial approach. Our selection of an orbitozygomatic approach for three cases was due to large cyst size or suprasellar extension, with a need for extension of the surgical field cranially. Our data support the safety of this technique; recognizing the limitations of our small sample size, none of the three patients who underwent orbitozygomatic approaches to cyst resection experienced intra- or postoperative complications. Additionally, all patients in our cohort had postoperative mRS scores of 0 or 1, further indicating that surgical resection is well tolerated. These low rates of morbidity for the surgical resection of pediatric RCC aligns with the existing literature [6, 8, 9, 11, 13, 14, 16, 17].

The secretory nature of RCC epithelium lends itself to recurrence following surgical treatment. Rates throughout the literature vary significantly due to differences in follow-up duration. Lower relapse rates are reported in case series with mean observation periods under three years, while relapse rates nearing 50% are seen in case series with follow-up periods extending beyond four years.[12] Recurrence usually occurs within the first 5–6 years following surgery [5, 13–15], and there does not appear to be an association between surgical approach and recurrence rate. Our 32% rate of recurrence requiring surgery over a mean > 5-year follow-up, and 47.8% 5-year progression free survival aligns with this prior literature and highlights the need for ongoing surveillance in this patient population. In patients with recurrent lesions, parameters for reoperation were similar to those for initial surgical resection, with recurrent surgery well-tolerated and most patients requiring only a single reoperation. This series includes patients who underwent surgical resection, but does not encompass the natural history and expectant management of the vast majority of pediatric pituitary cysts. These results represent our single institution experience and are not necessarily representative of the patient population and treatment approaches at other centers. Furthermore, we did not prospectively record the extent of cyst wall resection, which may be a predictive factor for postoperative endocrinopathy. Larger prospective, multicenter studies can be performed to increase power and generalizability.

## Conclusion

Similar to adults, pediatric RCCs may demonstrate rapid growth and can cause symptoms due to local mass effect. Surgical management of symptomatic or growing pediatric RCCs via cyst fenestration or partial resection of the cyst wall can be performed safely, with good neurologic outcomes. However, surgery carries a nontrivial risk of endocrinologic deficits. Long-term follow up of this patient population is needed due to high recurrence rates.
